# Oral Microbiota and Inflammatory Bowel Diseases: Detection of Emerging Fungal Pathogens and Herpesvirus

**DOI:** 10.3390/biomedicines13020480

**Published:** 2025-02-15

**Authors:** Manoel Marques Evangelista Oliveira, Letícia Bomfim Campos, Fernanda Brito, Flavia Martinez de Carvalho, Geraldo Oliveira Silva-Junior, Gisela Lara da Costa, Tatiane Nobre Pinto, Rafaela Moraes Pereira de Sousa, Rodrigo Miranda, Rodolfo Castro, Cyrla Zaltman, Vanessa Salete de Paula

**Affiliations:** 1 Laboratory of Taxonomy, Biochemistry and Bioprospecting of Fungi, Oswaldo Cruz Institution (IOC), Oswaldo Cruz Foundation (Fiocruz), Rio de Janeiro 21040-360, RJ, Brazil; gisela.costa@fiocruz.br (G.L.d.C.); tatianepinto@aluno.fiocruz.br (T.N.P.); r-prado-miranda@bol.com.br (R.M.); 2 Laboratory of Molecular Virology and Parasitology, Oswaldo Cruz Institution (IOC), Oswaldo Cruz Foundation (Fiocruz), Rio de Janeiro 21040-360, RJ, Brazil; leticia_bonfim1998@hotmail.com (L.B.C.); rafaelamoraesfiocruz@gmail.com (R.M.P.d.S.); 3 Department of Periodontology and Diagnostics and Therapeutics, School of Dentistry, Rio de Janeiro State University, Rio de Janeiro 20551-030, RJ, Brazil; fernanda.brito.s@hotmail.com (F.B.); silvajuniorgo@gmail.com (G.O.S.-J.); 4 Laboratory of Epidemiology of Congenital Malformations, Oswaldo Cruz Institution (IOC), Oswaldo Cruz Foundation (Fiocruz), Rio de Janeiro 21040-360, RJ, Brazil; flavia.carvalho@ioc.fiocruz.br; 5 Post-Graduation Programme in Genetics, Federal University of Rio de Janeiro, Rio de Janeiro 21941-617, RJ, Brazil; 6 Sergio Arouca National School of Public Health, Oswaldo Cruz Foundation, Rio de Janeiro 21040-360, RJ, Brazil; rodolfoalcastro@gmail.com; 7 Institute of Collective Health, Federal University of the State of Rio de Janeiro, Rio de Janeiro 21941-598, RJ, Brazil; c.zaltman@gmail.com

**Keywords:** oral microbiome, emergent fungi, chronic intestinal inflammation, human herpesvirus, oral dysbiosis

## Abstract

**Background/Objectives:** Ulcerative colitis (UC) and Crohn’s disease (CD) are the usual clinical forms of inflammatory bowel disease (IBD). Changes in the oral microbiota, especially the presence of emerging fungi and herpesviruses, have been shown to worsen the clinical aspects of IBD. The aim of this study was to screen for emerging pathogens in the oral yeast microbiota and the presence of herpesvirus in IBD patients. **Methods:** Oral swabs of seven UC or CD patients were collected. The samples were plated on Sabouraud Dextrose Agar and subcultured on CHROMagar Candida and CHROMagar Candida Plus. Polyphasic taxonomy was applied and identified using molecular tools, such as MALDI-TOF MS and ITS partial sequencing. Multiplex qPCR was used to identify the herpesvirus. Results: The mean age was 38.67 ± 14.06 years, 57.14% were female, and two had diabetes. The CD patients presented with *Rhodotorula mucilaginosa, Candida orthopsilosis* and *Kodamaea jinghongensis*, while the UC patients presented with *Cutaneotrichosporon dermatis*, *Candida glabrata*, *Candida lusitanea* and *Candida tropicalis*. Two UC individuals had at least one herpesvirus. In the first individual, a co-detection of Herpes Simplex Virus 1 (HSV-1) and *C. lusitaniae* was observed. The second presented with co-infections of Epstein–Barr virus (EBV), Human Herpesvirus 7 (HHV-7) and *C. tropicalis*. **Conclusions:** We identified rarely described yeasts and co-infections in IBD patients, highlighting the need to identify emerging pathogens in the oral microbiota, as they may contribute to opportunistic infections.

## 1. Introduction

Inflammatory bowel diseases (IBD), which include Crohn’s disease (CD) and ulcerative colitis (UC), are a group of conditions characterized by chronic inflammation at various sites in the gastrointestinal tract [1]. Their etiology and pathogenesis are not well established, but it is speculated to be multifactorial and involves genetic, environmental, immune and microbial factors. The microbiota, which includes a constellation of archaea, bacteria, fungi, viruses, protists and helminths, was associated with IBD susceptibility, clinical aspects, disease activity or remission, and response to treatment [2].

Oral and bowel involvement in CD patients is highly associated with significant reductions in bacterial diversity in both microbiomes, with a decrease in beneficial bacteria, such as *Faecalibacterium prausnitzii*, and an increase in potentially pathogenic species, such as *Escherichia coli* [3,4]. However, the oral cavity is the second largest microbiota in the human body, being colonized by bacteria, but also by fungi and viruses that can lead to the worsening of IBD [5].

The oral cavity is a unique ecosystem that comprises a wide range of habitats, including the teeth, gingival sulcus, tongue, cheeks, tonsils, and the hard and soft palates. The colonization of the human oral microbiome mostly occurs on tooth surfaces, which leads to biofilm formation that is associated with dental plaque [5,6]. This oral microbiota is, in broad terms, similar in all humans, although each person has a characteristic “finger-print” that is unique [7].

Most of the common yeasts found in healthy oral cavities are from the *Candida* group, with *C. albicans* being predominantly found in the superior gastric tract and along the gut [8,9,10]. In addition, representatives of the *Basidiomycota* and *Ascomycota* clades can still be found [10]. Rivera et al. (2019) measured the profile of yeasts isolated in healthy individuals and 52.0% were identified as *C. albicans*, 43.5% non-*C. albicans* (*C. parapsilosis, C. dubliniensis, C. glabrata, C. tropicalis* and *C. intermedia*) and the least isolated species were *Geotrichum candidum* (1.5%) and *Rhodotorula mucilaginosa* (1.5%) [11].

Although these and other fungal species are part of the healthy oral microbiota, they can cause opportunistic infections that are generally associated with an inflammatory response from the host, which can impact the quality of life of individuals [12]. Above all, some minor yeast species can be found in the oral tract under certain circumstances and can reflect some health and disease statuses [13]. Three species of the genus *Rhodotorula* (*R. mucilaginosa, R. minuta* and *R. glutinis*), commonly described as opportunistic fungi in immunodeficient patients, were found in oral cavity of patients with colorectal cancer [14].

*Kodamaea* are considered an emergent pathogen, with *K. ohmeri* being the most common pathogen present in nosocomial environments, reported as a causative agent of fungemia mainly in neonatal, elderly and immunocompromised patients [15,16]. In Brazil, saliva and oropharyngeal candidiasis samples from HIV-positive patients presented with *C. albicans* (51.56%), non-*C. albicans* species (43.73%), *Trichosporon mucoides* (3.12%) and *K. ohmeri* (1.56%) [17].

In IBD patients, changes in the oral microbiota, especially the presence of emerging fungi, can be related to disease progression [18]. Studies have shown a significant change in the fungal microbiota of UC or CD patients in comparison to healthy individuals [19,20,21,22,23]. *Trichosporon* is a common fungus present in the mouth of healthy individuals; however, it is being pointed to as a potential emergent fungus in CD patients [24,25].

In addition, according to Mouzan et al. (2017), the fungal microbiome of children with aggravated CD in their mucosal tissues includes the phyla *Basidiomycota* and *Ascomycota*, where there is population-level growth of *Basidiomycota* fungi, such as *Psathyrella*, and a decrease in *Ascomycota* fungi, such as the order *Helotiales* [23]. Therefore, it is important to investigate fungi, especially the presence of emerging fungi in the oral microbiota, to characterize risk factors in individuals with IBD.

As previously mentioned, the oral microbiota is also composed of viruses that are commonly found in this cavity, which are called the oral virome. They form a robust ecosystem with members that are capable of infecting human cells as well as bacterial, and may interfere with the health of the host [5,26]. The most common viruses that infect humans present in the oral microbiota are Human Papilloma Virus (HPV) and Human Herpesvirus (HHV) [27].

The presence of herpesviruses in mucous membranes has been associated with worsening of the inflammatory condition and progression of IBD [28]. Although rare, Herpes Simplex Colitis is associated with exacerbation of UC in immunosuppressed patients [29]. Leal et al. (2022) reported the presence of an opportunistic coinfection by Cytomegalovirus (CMV or HHV-5) and HSV-2 in a steroid/immunomodulator refractory UC patient, leading to the worsening of the clinical aspects and the need for a colectomy even after antiviral treatment [30].

The prevalence of Epstein–Barr virus (EBV or HHV-4) and CMV were higher in UC and CD patients when compared to healthy individuals, and the frequency of herpesviruses also increases with worsening intestinal disease [18]. Altunal et al. (2023) reported that CMV-positive UC patients had a higher frequency of steroid resistance, longer disease duration, longer remission and longer length of hospital stay [31]. All these data show that herpesvirus infections in individuals with IBD may be a risk factor for worsening of the clinical picture.

Although the importance of fungal gut microbiome dysbiosis for IBD has been elucidated, changes in the oral microbiota and their impacts are poorly reported. Furthermore, the risk of herpesvirus infection in UC patients is known, but there was no evidence until now on the impact of coinfection with herpesviruses and emerging fungi in individuals with IBD. Therefore, the main objective of this study was to investigate the presence of fungi and herpesviruses in the oral microbiota of patients with IBD to identify potential risk factors.

## 2. Materials and Methods

### 2.1. Sample Collection and Isolation of Yeast

This is a descriptive cross-sectional study, approved by the ethics committee (CAAE: 54235021.7.0000.5259). Informed consent was obtained from all subjects involved in this study, and samples were collected between 25 October 2022 and 25 November 2022. In total, 7 patients with inflammatory bowel disease (ulcerative colitis or Crohn’s disease) treated by Crohn’s disease outpatient clinic were included. Oral swabs were inserted into a tube with sterile saline solution and then passed through the entire oral cavity for 2 to 3 s and then placed into 5 mL tubes containing 2 mL of a 0.9% saline solution. The samples were then taken to the laboratory within 30 min of the samplings and processed the analysis.

**Figure 1:**
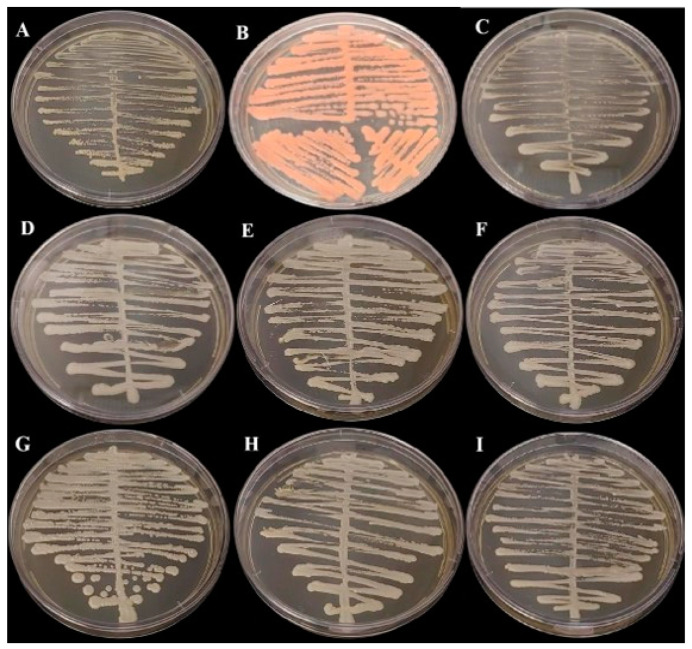
Growth in Sabouraud Dextrose Agar Medium (BD Difco) incubated at 35 °C for 48 h: (**A**) *Cutaneotrichosporon dermatis*, (**B**) *Rhodotorula mucilaginosa*, (**C**) *Candida glabrata*, (**D**) *Candida orthopsilosis*, (**E**) *Kodamaea jinghongensis*, (**F**) *Candida lusitanea*, (**G**) *Kodamaea jinghongensis,* (**H**) *Candida orthopsilosis* and (**I**) *Candida tropicalis*.

**Figure 2:**
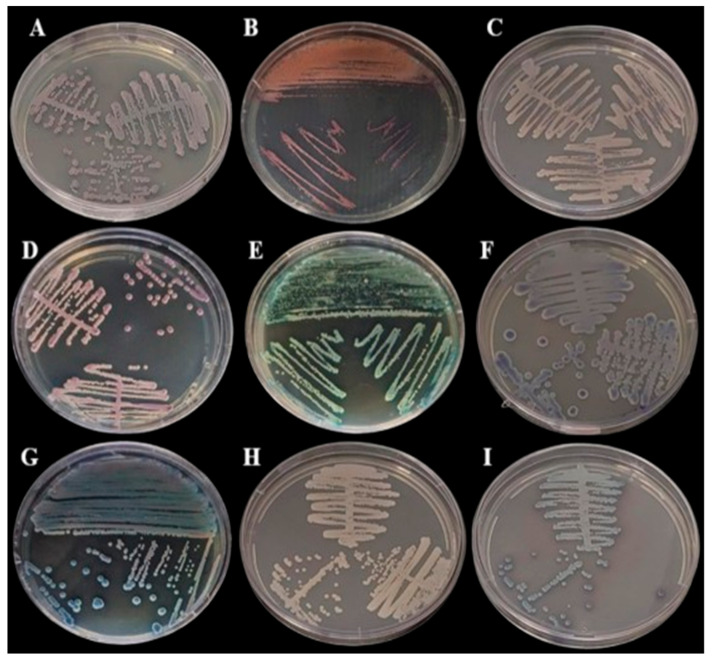
Growth in BDTM CHROMagar^TM^ Candida Medium (BD Difco) incubated at 35 °C for 48 h: (**A**) *Cutaneotrichosporon dermatis,* (**B**) *Rhodotorula mucilaginosa*, (**C**) *Candida glabrata*, (**D**) *Candida orthopsilosis*, (**E**) *Kodamaea jinghongensis,* (**F**) *Candida lusitanea*, (**G**) *Kodamaea jinghongensis,* (**H**) *Candida orthopsilosis* and (**I**) *Candida tropicalis*.

Samples were streaked onto Sabouraud Dextrose Agar (BD Difco^TM^, Franklin Lakes, BD, USA) and incubated at 30 °C for 48 h for morphological assessments. All samples presenting with growth on the SDA medium (Figure 1) were then subcultured onto CHROMagar Candida (BD Difco^TM^, Franklin Lakes, BD, USA) and CHROMagar Candida Plus (CHROMagar^TM^, Saint-Denis, France) (Figure 2) and colonies were interpreted according to the manufacturer’s instructions.

Polyphasic taxonomy was applied with morphologic and phenotypic tests for all isolates obtained and identified using molecular tools, such as MALDI-TOF MS and partial sequencing of the ITS region (Pinto et al., 2022 [32]). Fungal identification at the species level by MALDI-TOF MS was carried out as previously described by Pinto et al. (2022) [32]. Briefly, 10^6^ yeast cells were transferred from the culture plate (c.a. 1g) to a 500 μL tube containing 20 µL of 70% formic acid in water (*v*/*v*) and mixed with 10 µL of acetonitrile. The samples (1 µL) were then spotted onto a stainless Bruker MALDI-TOF MS plate (Bruker, Billerica, MA, USA) and covered with 1 µL of a α-cyano-4-hydroxycinnamic acid (CHCA, Fluka, Buchs, Switzerland) used as the matrix. Each sample was analyzed in triplicate. Samples were air-dried at room temperature prior to spectra acquisition. Results are expressed as log values ranging from 0 to 3, where values of 1.7 are generally used for reliable genus identification and values of 2.0 are used for reliable species identification (Stevenson et al., 2010 [33]).

The genomic DNA from the yeast, obtained by the extraction of colonies grown on SDA, was performed using Gentra^®^ Puregene^®^ Yeast and G+ Bacteria kits (Qiagen^®^, Germantown, MD, USA) according to the manufacturer’s recommendations. Briefly, the amplification of the ITS1-5.8S-ITS2 ribosomal DNA region was performed in a final volume of 50 µL containing 100 ng of DNA and 10 µL of each primer (Invitrogen^TM^ São Paulo, Brazil), ITS1 (5′ TCCGTAGGTGAACCTGCGG 3′) and ITS4 (5′TCCTCCGCTTATTGATATGC 3′) (Lindsley et al., 2001 [34]). PCRs were performed employing a Veriti Applied Biosystems thermocycler at an annealing temperature of 58 °C. Automated sequencing was performed using the Fundação Oswaldo Cruz sequencing platform (PDTIS/FIOCRUZ, Rio de Janeiro, Brazil). The sequences were edited using CodonCode Aligner (Genes Code Corporation, Ann Arbor, MI, USA), and phylogenetic analyses were performed using the Blast software (version 2.16.0) for comparison with sequences deposited in the NCBI/GenBank database.

### 2.2. Phylogenetic Analyses

Evolutionary analyses were conducted in MEGA X [35]. The phylogenetic relationships between the sample isolates and the reference strains from ITS sequences were performed by the Neighbor Joining method [36]. The percentages of replicate trees in which the associated taxa clustered together in the bootstrap test (1000 replicates) are shown next to the branches [37]. The evolutionary distances were computed using the Maximum Composite Likelihood method [38] and are in the units of the number of base substitutions per site.

### 2.3. Herpesvirus qPCR

For the analysis of the presence and quantification of the viral load of herpesviruses, the samples were extracted using the “QIAamp DNA Mini Kit” (QIAGEN, Hilden, Germany). Then, the samples were analyzed using real-time PCR (qPCR) through the commercial kit AgPath-IDTM One-Step RT-PCR Kit (Life Technologies, Carlsbad, CA, USA). A multiplex real-time PCR (qPCR) was performed for each herpesvirus subfamily (Alphaherpesvirus, Betaherpervirus and Gammaherpesvirus) according to previously established protocols, and synthetic curves were used to quantify the viral load [39,40,41,42].

### 2.4. Data Analisis

Exploratory data analysis (EDA) was used to better observe the results. Initially, the data was organized and cleaned, removing incomplete records and inconsistencies. Then, measures of central tendency (mean) and measures of dispersion (standard deviation) were applied, in addition to establishing frequencies represented in percentages, to summarize the main characteristics of the data. This exploratory approach allowed a deeper understanding of the dataset.

## 3. Results

### 3.1. Characteristics of Patients

The basic characteristics of the individuals analyzed in this study are the mean age of the patients, which was 38.67 ± 14.06 years, and the sex, where 57.14% (4/7) were female. In addition, the mean body mass index (BMI) was 22.46 ± 5.78, two individuals had diabetes and no patients were smokers. Among the IBD group, 57.14% (4/7) had UC and 42.86% (3/7) had CD, and the average disease duration was 13.7 ± 5.7 years. Five of these patients showed disease remission (three with UC and two with CD), three of these individuals received biological medication (one with active disease and two in remission), and finally, no patients received corticosteroids. In this study, 85.7% (6/7) of the participants had some level of periodontal disease and the average number of teeth was 20.3 ± 10.9.

### 3.2. Detection and Characterization of Fungi

In the polyphasic taxonomy, we observed the presence of three genres of yeast in the oral microbiome, as the partial sequencing of the ITS regions identified species of the genera *Kodamaea, Trichosporon, Rhodotorula* and *Candida*. The Figure 1 and Figure 2 show, respectively, the growth of fungi in Sabouraud Dextrose Agar Medium (BD Difco, Franklin Lakes, NJ, USA) and in BDTM CHROMagar^TM^ Candida Medium (BD Difco, Franklin Lakes, NJ, USA).

**Figure 3:**
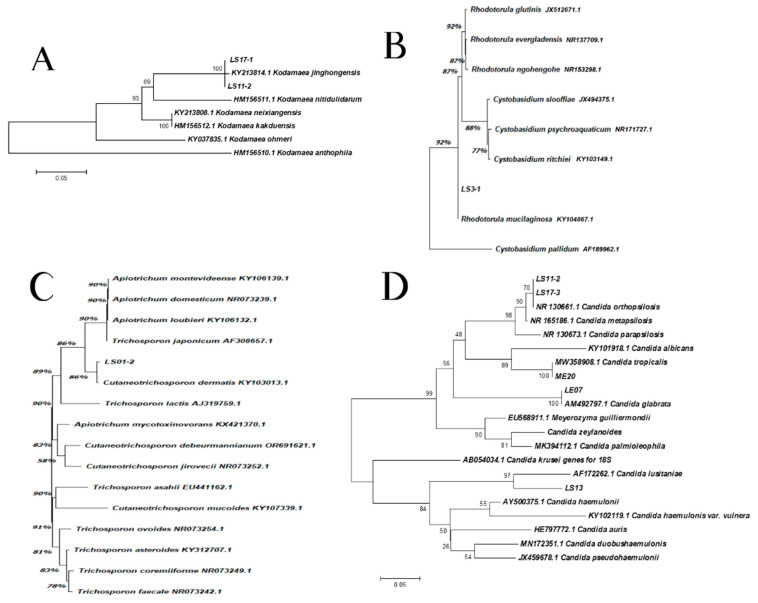
The phylogenetic relationships between the isolates of samples with reference strains inferred from ITS sequences. (**A**) *Kodhamaea* sp.: this analysis involved eight nucleotide sequences, and a total of 263 positions were obtained in the final dataset. (**B**) *Rhodotorula* sp.: this analysis involved nine nucleotide sequences, and a total of 602 positions were obtained in the final dataset. (**C**) *Trichosporon* sp.: this analysis involved 16 nucleotide sequences and a total of 560 positions were obtained in the final dataset. (**D**) *Candida* spp.: this analysis involved 22 nucleotide sequences and a total of 92 positions were obtained in the final dataset.

Isolation, sequencing and alignment of the ITS region showed a total correspondence between LS11-2, LS17-1 and *K. jinghongensis* (KY213814.1) (Figure 3A). LS3-1 showed a significant correspondence to *R. mucilaginosa* (CBS 11029) (Figure 3B), which is highly related (92%) to *Cystobasidium pallidum*. Isolate LS01-2 showed a significant correspondence to *Cutaneotrichosporon dermatis* (86%) (KY103013.1) (Figure 3C), which is significantly related to the clade composed of *Apiotrichum montevideense* (KY106139.1), *A. domesticum* (NR073239.1), *A. loubieri* (106132.1) and *T. japonicum* (90%) (AF308657.1). Isolates LS11-1 and LS17-3 revealed 70% correspondence to species *C. orthopsilosis* (NR130661.1) (Figure 3D). However, the group formed by isolates LS11-1, LS17-3 and *C. orthopsilosis* were 90% correspondent to *C. metapsilosis* (NR130673.1) (Figure 3D). Isolate ME20 revealed a full correspondence to *C. tropicalis* (MW358908.1), while isolate LE07 corresponded in the same pattern to *C. glabrata* (AM492797.1) (Figure 3D). Isolate LS13 was 97% correspondent to *C. lusitaniae* (AF172262.1) (Figure 3D).

In this study, when the fungi detected according to the etiology of IBD were observed (Table 1), it was found that patients with CD presented with *R. mucilaginosa*, *C. orthopsilosis* and *K. jinghongensis*, while individuals with UC presented with *C. dermatis*, *C. glabrata*, *C. lusitanea* and *C. tropicalis*.

### 3.3. Detection and Characterization of Herpesvirus

Of the samples analyzed, two (28.57%) presented with infection by at least one herpesvirus (Table 2). In sample LS13, the presence of HSV-1 was detected (viral load 1.07 × 10^4^). In addition, this patient had UC, and *C. lusitaniae* was detected. While sample ME20 presented with a co-infection of EBV (viral load 7.40 × 10^4^) and HHV-7 (viral load 2.26 × 10^5^), the patient also had UC, and *C. tropicalis* was detected.

## 4. Discussion

In this study, different fungal species were observed according to IBD types (CD and UC). *C. dermatis* was identified in an isolate from a UC patient, representing an important yeast associated with rare and opportunistic infections in immunocompromised and even pediatric patients [43,44,45]. Furthermore, studies show a potential risk of opportunistic infections related to this fungus, due to antifungal resistance and its ability to produce important virulence factors [46,47]. However, there are no studies in IBD patients. In addition to yeasts, the presence of herpesviruses was identified in the oral microbiota of two UC patients.

Many microorganisms present in the human microbiome may serve as a physiological indicator of good health or a potential inflammatory process. The oral mycobiome is predominantly formed by several genera, including *Candida, Cladosporium, Saccharomyces, Fusarium, Aspergillus, Aureobasidium* and *Cryptococcus* [48,49].

*R. mucilaginosa*, found in this study, is an opportunistic and emerging agent, which can cause infections mainly in immunocompromised patients, and has already been reported in the oral microbiota of patients with colorectal cancer [14,50,51,52,53,54]. Furthermore, in oral *R. mucilaginosa* isolates from patients with chronic kidney disease, all isolates presented with antifungal resistance to azoles and the ability to form biofilms [55]. Therefore, colonization by *R. mucilaginosa* may be a risk factor for serious infection in patients with chronic diseases, such as CD.

*K. jinghongensis* was found to be present in an oral isolation from a CD patient. *K. jinghongensis* is a yeast species first described by Gao et al. (2017) and to date, there are no studies on the impact of *K. jinghongensis* infection on human health [56,57]. *Kodamaea* is known to be an important opportunistic yeast genus, with *K. ohmeri* being the most reported pathogen for fungemia, and this species was detected in oral samples from patients with head and neck cancer [15,16,58]. Therefore, further studies are needed to evaluate the impact of *K. jinghongensis* infections, especially in individuals with IBD.

The most common yeasts found in the oral microbiota are from the *Candida* genus, and they can switch from commensal to pathogenic depending on host homeostasis [10]. In this study, a *C. orthopsilosis* isolate was found in one CD patient and *C. glabrata, C. lusitanea* and *C. tropicalis* were found in UC individuals. *Candida* species are the most common agents of infection in IBD patients, but the influence of *C. orthopsilosis* and *C. lusitanea* on IBD is not well established [59,60,61].

The presence of HSV-1 was identified, which has been associated with severe necrotizing tonsillitis and rare cases of Herpes Simplex Colitis, leading to the exacerbation of UC [29]. Additionally, a coinfection between EBV and HHV-7 was identified. EBV infection is more frequent in individuals with IBD and is associated with more severe cases of the disease [18]; however, its association with other herpesviruses, such as HHV-7, can further aggravate the inflammatory condition of these individuals [62,63]. Therefore, identifying possible infections and/or reactivations of herpesviruses in patients with IBD may be important for the differential diagnosis of these individuals. Herpesvirus has been detected in the oral cavities of immunocompromised patients, but the presence of fungal species remains rare [64,65,66,67].

Patients with a viral load for herpesvirus were positive for the *Candida* group (*C. lusitaniae* and *C. tropicalis*). In the literature, a mutualistic relationship between Herpes Simplex (HSV-1 and HSV-2) and *C. albicans* is reported, where the viruses can be included in the fungal biofilm, becoming more resistant to pharmacological treatments (acyclovir) [68,69]. In addition, the presence of Herpes Simplex infection increases the adherence and initial formation of *C. albicans* biofilms [70,71], indicating that the interaction between these different species is complex and can influence the dynamics of infection and consequently the health of the individual. However, studies are lacking in determining the association between herpesviruses and other species of *Candida*, as well as other types of herpesviruses besides Herpes Simplex.

Studies have shown that alterations in the fungal microbiota in patients with IBD, especially the presence of emerging fungi, have been shown to be a factor in the worsening of the clinical condition of these individuals [18,19,20,21,22,23]. Furthermore, the presence of herpesviruses in mucous membranes has been associated with the worsening of the inflammatory condition and exacerbation of IBD [28].

Although CD and UC are both classified as IBD, they have distinct pathogeneses. CD is characterized by segmented inflammation that can affect any part of the gastrointestinal tract, being most common in the ileum, while UC is characterized by continuous inflammation that is predominantly in the colon and rectum [72]. The literature describes that the oral microbiome may present with significant differences between the types of IBD. Xun et al. (2018) described that, in individuals with CD, an enrichment of species from the *Veillonellaceae* family and a decrease in *Neisseriaceae* and *Haemophilus* could be observed. In individuals with UC, an enrichment of the *Streptococcaceae* and *Enterobacteriaceae* family and a decrease in *Lachnospiraceae* and *Prevotella* in the oral microbiota were observed [73]. These differences in the oral microbiomes can be explained by the distinct pathogenesis of IBD and may also influence the development of the disease, since immune responses can vary considerably between CD and UC. However, to date, studies have focused on bacterial differences in the oral microbiome. Further studies are needed to investigate whether there are also differences in the presence of fungi and viruses in the oral cavity of patients with CD and UC.

The severity of the pathogens identified may vary, as they are influenced by the individual’s immune status. In general, opportunistic pathogens, such as the fungal species identified in this study, can cause a variety of clinical manifestations and can lead to severe invasive infections, especially in immunocompromised individuals. In addition, some fungal species identified have already been associated with antifungal resistance, which may contribute to greater disease severity and therapeutic challenges.

The infection process is complex and multifactorial, but it generally begins with immunological alterations and the establishment of a proinflammatory environment, which favors dysbiosis of the human microbiome and alters the integrity of mucosal barriers. In other words, a series of conditions occur that will favor the proliferation of microorganisms and the establishment of a local or systemic infection.

In addition, the herpesviruses identified in our study are known for their ability to establish latency and reactivate under conditions of immunosuppression. This reactivation can contribute to the severity of the disease, either through direct effects or by modulating the immune response, facilitating infection by other opportunistic pathogens.

Investigating the presence and co-occurrence of herpesvirus and fungal species in the oral cavity of immunocompromised patients is crucial, as these infections may complicate the clinical management of such patients. Understanding the interactions between viral and fungal pathogens could reveal novel insights into their combined impacts on immune suppression, potentially informing better diagnostic and therapeutic approaches. Additionally, identifying fungal species in these contexts could help anticipate and prevent opportunistic infections, improving patient outcomes and quality of life.

## 5. Conclusions

It is crucial to investigate the presence of herpesviruses and fungi, particularly emerging fungal species, within the oral microbiota to better understand the risk factors for aggravating IBD in individuals with this disease. To our knowledge, this is the first study to document the presence of the emerging fungi *K. jinghongensis* and *C. dermatis* in the oral microbiota of IBD patients. Additionally, we report the presence of rarely described yeasts, including *R. mucilaginosa, C. orthopsilosis* and *C. lusitaniae*, as well as the coexistence of HSV-1/*C. lusitaniae* and EBV/HHV-7/*C. tropicalis* in UC patients. These findings underscore the importance of identifying emerging pathogens within the oral microbiota and potential opportunistic infections in individuals with chronic intestinal inflammation. The presence of these pathogens could influence inflammatory processes and, consequently, the prognosis of these patients, highlighting the need for continued research into the microbiota’s role in IBD development, progression and management.

## Figures and Tables

**Table 1:** Detection and characterization of fungi according to type of inflammatory bowel disease.

Isolate	Sex	Age	Type of IBD	Species of Fungi
LS01-2	Male	51	UC	*Cutaneotrichosporon dermatis*
LS03-1	Female	49	CD	*Rhodotorula mucilaginosa*
LE07	Female	63	UC	*Candida glabrata*
LS11-1/LS11-2	Male	40	CD	*Candida orthopsilosis*/*Kodamaea jinghongensis*
LS 13	Male	31	UC	*Candida lusitanea*
LE17-1/LS17-3	Female	21	CD	*Kodamaea jinghongensis*/*Candida orthopsilosis*
ME20	Female	32	UC	*Candida tropicalis*

IBD: Inflammatory bowel disease; CD: Crohn’s disease; UC: Ulcerative colitis.

**Table 2:** Detection and quantification of samples with herpesvirus-positivity.

Sample	IBD	Viral Load			Fungi Detection
		HSV-1	EBV	HHV-7	
LS13	UC	1.07 × 10^4^	Undetermined	Undetermined	*C. lusitaniae*
ME20	UC	Undetermined	7.40 × 10^4^	2.26 × 10^5^	*C. tropicalis*

All study samples were analyzed for all human herpesviruses; this table shows only those with a detectable viral load. The others were undetermined (no viral load detected). EBV: Epstein–Barr virus; HSV-1: Herpes Simplex Virus 1; HHV-7: Human Herpesvirus 7; IBD: inflammatory bowel disease; UC: ulcerative colitis.

## Data Availability

The original contributions presented in this study are included in the article. Further inquiries can be directed to the corresponding authors.

## Abbreviations

The following abbreviations are used in this manuscript:

IBD	Inflammatory Bowel Disease
CD	Crohn’s Disease
UC	Ulcerative Colitis
HPV	Human Papilloma Virus
HHV	Human Herpes Virus
CMV	Cytomegalovirus
EBV	Epstein–Barr virus
SDA	Sabouraud Dextrose Agar
PCR	Polymerase Chain Reaction
qPCR	Real-time PCR
EDA	Exploratory Data Analysis
